# Venetoclax for Children and Adolescents with Acute Lymphoblastic Leukemia and Lymphoblastic Lymphoma

**DOI:** 10.3390/cancers14010150

**Published:** 2021-12-29

**Authors:** Amber Gibson, Adriana Trabal, David McCall, Sajad Khazal, Laurie Toepfer, Donna H. Bell, Michael Roth, Kris M. Mahadeo, Cesar Nunez, Nicholas J. Short, Courtney DiNardo, Marina Konopleva, Ghayas C. Issa, Farhad Ravandi, Nitin Jain, Gautam Borthakur, Hagop M. Kantarjian, Elias Jabbour, Branko Cuglievan

**Affiliations:** 1 Department of Pediatrics, The University of Texas MD Anderson Cancer Center, Houston, TX 77030, USA; AMTrabal@mdanderson.org (A.T.); DMcCall1@mdanderson.org (D.M.); LEParrott@mdanderson.org (L.T.); dabell@mdanderson.org (D.H.B.); mroth1@mdanderson.org (M.R.); cnunez@mdanderson.org (C.N.); Bcuglievan@mdanderson.org (B.C.); 2 Department of Pediatric Stem Cell Transplantation and Cellular Therapy, The University of Texas MD Anderson Cancer Center, Houston, TX 77030, USA; sjkhazal@mdanderson.org (S.K.); KMMahadeo@mdanderson.org (K.M.M.); 3 Department of Leukemia, The University of Texas MD Anderson Cancer Center, Houston, TX 77030, USA; nshort@mdanderson.org (N.J.S.); CDiNardo@mdanderson.org (C.D.); mkonople@mdanderson.org (M.K.); GCIssa@mdanderson.org (G.C.I.); fravandi@mdanderson.org (F.R.); njain@mdanderson.org (N.J.); gborthak@mdanderson.org (G.B.); hkantarjian@mdanderson.org (H.M.K.); ejabbour@mdanderson.org (E.J.)

**Keywords:** acute lymphoblastic leukemia, lymphoblastic lymphoma, venetoclax, Bcl-2 inhibitor, early precursor T-cell

## Abstract

**Simple Summary:**

Pediatric patients with relapsed or refractory acute lymphoblastic leukemia (ALL) or lymphoma (LBL) currently have unsatisfactory outcomes, and novel treatment options are needed. Venetoclax is approved for adult patients with several types of leukemia and is being investigated in the pediatric population. Here, we retrospectively reviewed the safety and efficacy of venetoclax for the treatment of ALL/LBL in the pediatric and young adult populations. The purpose of this study is to provide evidence that venetoclax is safe and effective to use in pediatric patients with ALL/LBL and should be considered in both the relapsed and upfront settings.

**Abstract:**

Venetoclax is approved for adult patients with chronic lymphocytic leukemia and acute myeloid leukemia. Expanding its use to the pediatric population is currently under investigation, but more robust data are needed. We retrospectively analyzed the safety and efficacy of venetoclax in children/AYA with ALL/LBL. We identified 18 patients (T-cell ALL, *n* = 7; T-cell LBL, *n* = 6; B-cell ALL, *n* = 5) aged 6–22 years. No new venetoclax safety signals were identified; the most common toxicity was myelosuppression. No deaths occurred within 30 days from the start of the therapy. A mean of 2.6 (range 0–8) prior lines of therapy were given. The mean duration of venetoclax was 4.06 months (range 0.2–24.67 months). Complete remission was achieved in 11 (61%) patients. Of the eight patients who remain alive, four are continuing on venetoclax combination therapy, and four proceeded to hematopoietic stem cell transplantation. Three patients who initially achieved CR, later relapsed, and are deceased. Nine patients are deceased, and one patient was lost to follow-up. Overall survival is 9.14 months (range 1.1–33.1), and progression-free survival is 7.34 months (range 0.2–33.1). This is the largest cohort of pediatric/AYA patients who received venetoclax for ALL/LBL. Our data support the consideration of venetoclax-based regimens in pediatric patients with R/R ALL/LBL and its investigation as upfront therapy for T-cell ALL/LBL.

## 1. Introduction

Pediatric acute lymphoblastic leukemia (ALL) and lymphoblastic lymphoma (LBL) now have remarkable outcomes, with event-free survival (EFS) >90% for B-cell ALL, 85–89% for T-cell ALL, and 80–85% for T-cell LBL patients [1,2,3,4,5,6]. Unfortunately, for patients with relapsed/refractory (R/R) disease, survival declines significantly after the first relapse and further with each subsequent relapse [1]. For B-cell ALL, relapse is seen in >10% of patients, and 2% of patients never achieve first remission due to refractory disease [7,8]. When focusing on T-cell ALL, seen in 15% of the pediatric ALL population, and the early thymic precursor (ETP) phenotype, the relapse rate is up to 18% [9,10,11]. Prognosis becomes especially poor for patients with relapsed T-cell ALL/LBL, with a dismal response to salvage therapy and a 3-year EFS <15% [12,13]. Such low survival for R/R disease, therefore, mandates the continued development of novel agents.

One such novel agent is venetoclax, a BH3 mimetic inhibitor of the anti-apoptotic protein B-cell lymphoma-2 (BCL-2), which restores cells’ apoptotic ability. The overexpression of BCL-2 family proteins is associated with disease progression and resistance to chemotherapy [14]. High levels of BCL-2 and/or BCL-2 “dependence” are observed in patients with follicular lymphoma, mantle cell lymphoma, diffuse large B-cell lymphoma, and adult leukemias, and BCL-2 inhibition in patients with these diagnoses has led to promising treatment results [15,16,17,18,19]. The BCL-2/BCL-XL inhibitors ABT-737, ABT-263, and venetoclax induce apoptosis in vitro and in vivo in ALL subsets, including KMT2A-rearranged and TCF3-HLF ALL leukemia xenografts and human lymphoid tumors that overexpress BCL-2 [18,20,21,22]. Recently, a casein kinase 2 inhibitor, silmitasertib, has shown promising synergism with venetoclax, particularly, in resistant B-cell ALL cell lines and xenografts [23]. Current studies show high efficacy in T-cell ALL adult and pediatric populations, but there remains sparse evidence of the effects of this combination in T-cell LBL patients [24,25,26,27]. A phase I study by Pullarkat et al. obtained promising results in 12 reported pediatric patients with ALL [27]. Venetoclax continues to be studied in the pediatric population, with current studies (NCT03236857 and NCT04029688) underway for relapsed pediatric B- and T-cell ALL [1,28].

Given the encouraging effects of venetoclax combinations with chemotherapy in patients with lymphoid malignancies and the sparsity of reported outcomes in T-cell LBL, and pediatrics/AYA in general, we retrospectively reviewed our institutional experience of venetoclax use in pediatric/AYA patients at The University of Texas MD Anderson Cancer Center and report the results regarding 18 patients ≤22 years of age with ALL/LBL who received venetoclax combination therapy.

## 2. Materials and Methods

After IRB approval, a retrospective chart review identified patients, 22 years old and younger, with a diagnosis of ALL/LBL, who received venetoclax at MD Anderson Cancer Center. Response criteria were established per the Revised Recommendations of the International Working Group Response Criteria in Acute Leukemia, and responses for lymphomas were according to the Malignant Lymphomas Imaging Working Group [29]. Complete remission (CR) was defined as disappearance of all clinical and/or radiologic evidence of disease, plus absolute neutrophil count (ANC) ≥1.0 × 10^3^/L, platelet count ≥100 × 10^3^/L, and bone marrow differential with <5% blasts. Complete remission without blood (or incomplete) count recovery (CRi) was defined as patients who met the criteria for CR, except for either residual neutropenia (ANC < 1.0 × 10^3^/L) or thrombocytopenia (platelet count < 100 × 10^3^/L) [30]. Minimal residual disease (MRD) was defined as multiparameter flow cytometry (FC) of bone marrow with less than 0.01% lymphoblastic cells. Venetoclax toxicities were graded per the Common Terminology Criteria for Adverse Events (CTCAE) version 5.0. Overall survival (OS) defined as the time in months from the start of venetoclax therapy to death, and progression-free survival (PFS) was defined as the time from the start of venetoclax administration until disease progression.

## 3. Results

### 3.1. Patient Characteristics

Eighteen patients were identified, 39% (*n* = 7) with T-cell ALL, 33% (*n* = 6) with T-cell LBL, and 28% (*n* = 5) with B-cell ALL, aged 6–22 years (median, 20). Of these patients, 44% had received >3 prior therapies (*n* = 8), 33% one prior therapy (*n* = 6), and 22% (*n* = 4) upfront venetoclax combination therapy. The mean number of prior therapies received was 2.6 (range 0–8). A history of prior HSCT was noted in 28% of the patients (*n* = 5). As shown in Table 1: the five identified B-cell ALL patients were heavily pretreated, all received prior CD19 or CD22 directed Chimeric Antigen Receptor (CAR) T cell therapy, one patients received a dual CD19/CD22 CAR, and one received blinatumomab. The patients’ prior therapies, cytogenetics, and molecular studies are listed in Table 2.

### 3.2. Treatment

All patients received venetoclax in combination with conventional chemotherapy, as reported in Table 2. The combination therapy consisted of cyclophosphamide, vincristine, dexamethasone, doxorubicin, methotrexate, and cytarabine (hyperCVAD) treatment in 50% (*n* = 9) of patients. Concurrent cyclophosphamide, vincristine, and dexamethasone (CVD) were administered to 23% (*n* = 4) of the patients. The five additional patients received different backbone therapies, as shown in Table 2. Depending on the diagnosis, four patients received venetoclax as upfront therapy with HyperCVAD, plus or minus nelarabine and pegylated asparaginase (peg-ASP). The patients who received upfront venetoclax therapy were 18 years old or older and were involved in clinical trials. The standard adult AML dosing of 400 mg daily (or adult equivalent weight-based dosing), with a bioequivalent dose for patients receiving a concurrent CYP3A4 inhibitor, was given. Dose or duration reduction of venetoclax was required in 22% of patients due to persistent myelosuppression. Patients received a mean of 4.06 cycles (median, 1.63), with a mean of 9.5 days (median, 7) of venetoclax per cycle.

The most common toxicity was thrombocytopenia; 89% of the patients developed grade 4 thrombocytopenia, and 22% (*n* = 4) required dose and/or duration adjustment of venetoclax due to this toxicity. Grade 4 neutropenia occurred in 50% (*n* = 9) and grade 3 or 4 febrile neutropenia in 28% (*n* = 5) of the patients. Grade 3 hyperbilirubinemia was diagnosed in 22% (*n* = 4), and grade 3 or 4 sepsis in 28% (*n* = 5) of the patients. Additional grade 3 or 4 toxicities included one of the following: aspartate aminotransferase (AST) elevation, mucosal infection, lung infection, and disseminated intravascular coagulation, as indicated in Table 3. No clinically significant tumor lysis syndrome was seen. Importantly, no deaths occurred within 30 days of the start of venetoclax combination therapy, and no deaths were reported as associated with venetoclax.

### 3.3. Response

**Figure 1:**
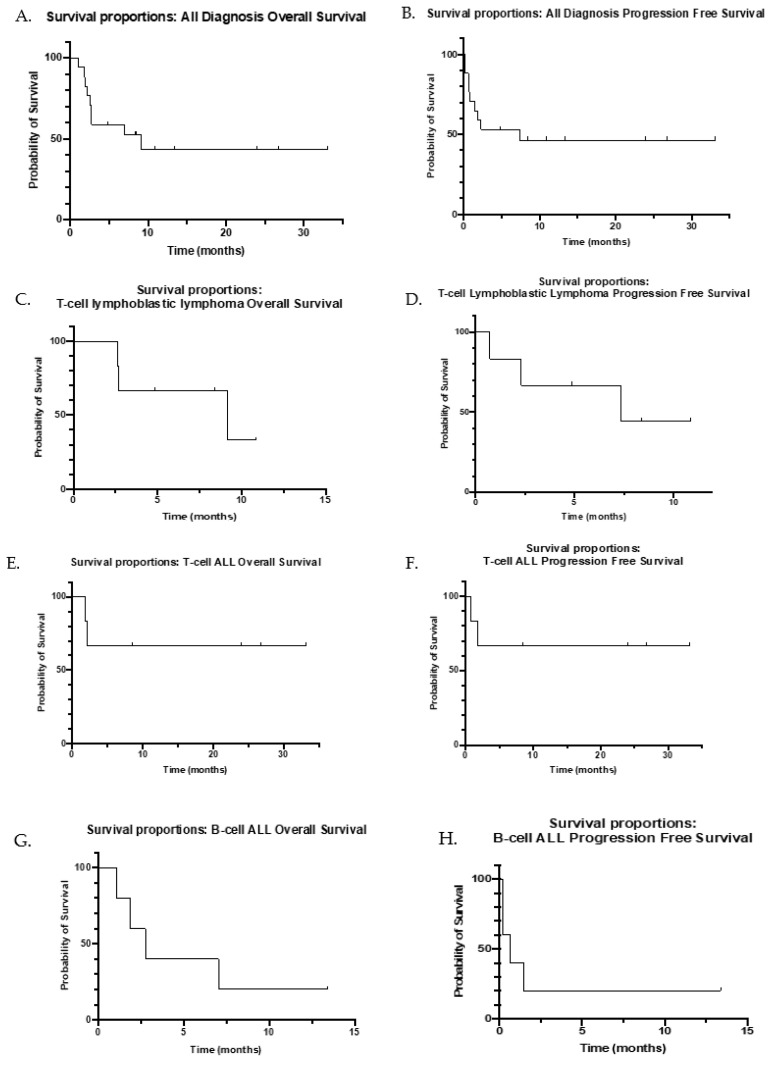
Kaplan–Meier curves for overall survival and progression-free survival. Survival proportions graphs from the start of venetoclax therapy to a major event, defined as progression or death. (**A**) Overall survival (OS) for all disease diagnoses. (**B**) Progression-free survival (PFS) for all disease diagnoses. (**C**) OS for T-cell LBL patients. (**D**) PFS for T-cell LBL patients. (**E**) OS for T-cell ALL patients. (**F**) PFS for T-cell ALL patients. (**G**) OS for B-cell ALL patients. (**H**) PFS for B-cell ALL patients.

Of the 18 patients, 61% responded (*n* = 11) with CR/CRi; 64% (*n* = 7) of the responders were treated for relapsed/refractory disease. Of the 11 patients who responded with CR/CRi, 5 had a diagnosis of T-cell ALL (1 with ETP), 5 of T-cell LBL, and 1 of B-cell ALL. Of the 13 patients with T-cell disease (ALL or LBL), 77% (*n* = 10) achieved CR/CRi, and 8 of these were patients with R/R disease. All responding patients obtained a uMRD negative status by FC or Deauville score of 2 or less. The mean number of cycles prior to response was 1.3 (median 1, range 1–4). Patients who had CR/CRi had a median of one prior line of therapy (range, 1–5), whereas patients with no response (NR) had a median of five prior lines of therapy (range, 1–8). To date, 80% (*n* = 8) of the evaluable patients who achieved CR/CRi remain alive, with a median follow-up time of 12.11 months (mean 16.22, range, 4.9–33.1). As shown in the Kaplan–Meier curves in Figure 1, the OS is 9.14 months (range 1.1–33.1), and the PFS is 7.34 months (range 0.2–33.1).

Patients with T-cell LBL had an OS of 9.14 months (range 2.63–10.86) and a PFS of 7.34 months (range 0.72–10.86). OS data have not yet been collected for patients with T-cell ALL. Patients with B-cell ALL had an OS of 2.76 months (range 2.76–13.4) and a PFS of 0.66 months (0.2–13.4).

**Figure 2:**
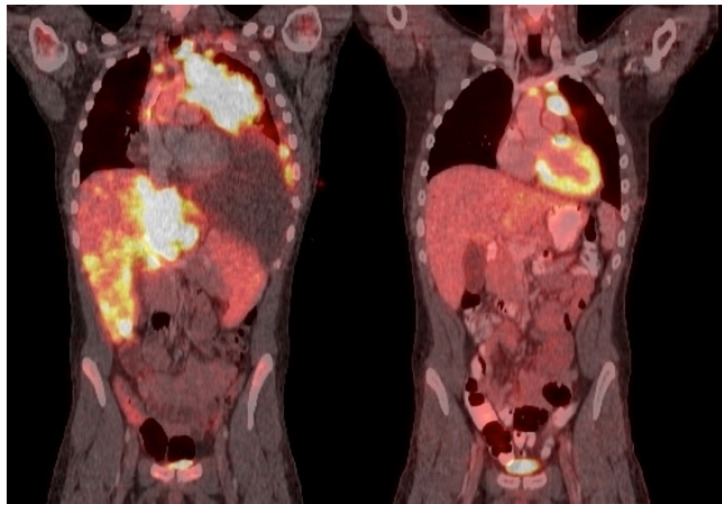
PETCT response of a patient with lymphoblastic lymphoma after one cycle of venetoclax combination therapy. PETCT response of patient 2 (see Table 2), with lymphoblastic lymphoma after one cycle of venetoclax combination therapy.

Response by diagnosis is summarized in Table 4. The investigation revealed that the CR of the six patients with T-cell LBL was 83% (*n* = 5) after a mean of 1.6 cycles (median 1, range 1–4) of venetoclax combination therapy. Sixty percent (*n* = 3) of these patients remain in remission, with ongoing venetoclax combination therapy and median follow-up time of 8.4 months (range 4.9–10.9). Two patients later progressed and died from their disease after initial CR. Patient 1, on Table 2 had NR to venetoclax combination therapy and died with disease. Patient 2 received four lines of prior therapy before receiving two cycles of venetoclax at 100 mg/day × 21 days, with a remarkable initial response after one cycle, as shown by PETCT, reported in Figure 2. Unfortunately, he then progressed after 2.5 months and died from progressive disease. Patient 3 had one prior line of therapy with a partial response and then achieved CR after one cycle of venetoclax combination therapy with 400 mg/day × 7 days. The patient remains in remission and has received cycle 7 of HyperCVAD+nelarabine+venetoclax therapy. Patient 4 had a mediastinal mass with marrow involvement of an aberrant T-cell population representing 1.4% of events similar to the immunophenotype identified on lymph node biopsy. She received 400 mg/day × 7 days of venetoclax combination therapy as frontline therapy, achieved CR after one cycle, and had uMRD after four cycles. Patient 5 received venetoclax combination therapy as frontline therapy at 400 mg/day × 7 days and achieved CR after one cycle. Venetoclax was discontinued after three cycles due to prolonged myelosuppression, but the patient remains in remission with a follow-up of 8.4 months. Patient 6 had a mediastinal and retroperitoneal mass as well as bone marrow involvement. She received hyperCVAD as frontline therapy but was given venetoclax combination therapy after progression and achieved CR with uMRD after one cycle. The duration of response was 7.3 months, but she ultimately progressed and died of disease.

Of the seven patients with T-cell ALL, 71% (*n* = 5) achieved CR after a mean of 1.2 cycles (median 1, range 1–2). The patients who achieved CR were three patients with R/R disease and two patients treated with upfront venetoclax combination therapy. The patients with R/R disease proceeded to HSCT; two of them are still alive with a median OS of 29.9 months, and one patient was lost to follow-up. Patient 8 (see Table 2), received venetoclax as upfront therapy for his diagnosis of T- cell ALL (HyperCVAD, nelarabine, and venetoclax) and achieved morphological remission with 0% BM blasts (from initial 90%) and FC with uMRD after one cycle of 400 mg/day for 7 days. He proceeded to receive induction, consolidation, intensification, and maintenance venetoclax combination therapy and remains in remission, on maintenance with venetoclax, receiving 400 mg/day × 3 days on 27-day cycles. Patient 9 with CNS3 relapse, received venetoclax after one line of prior therapy and achieved morphologic remission with 3% BM blasts and uMRD by FC 18 days after one cycle with 200 mg/day × 5 days of venetoclax (with concurrent voriconazole). He was recommended to proceed to HSCT but ultimately was lost to follow-up. Patient 10 with ETP ALL received venetoclax for refractory disease in his BM and extramedullary sites with bilateral shin and testis involvement. He obtained morphologic remission after one cycle of venetoclax 100 mg/day × 11 days with gemtuzumab. Then, he presented with uMRD after an additional 100 mg/day × 6 days with methotrexate. He was maintained on venetoclax-based consolidation to bridge to HSCT. He remains in remission, with a follow-up of 33.1 months. Patient 12 received frontline venetoclax combination therapy and achieved CR with uMRD after two cycles of 400 mg/day × 7 days. He proceeded to HSCT and remains in remission, with a follow-up duration of 8.5 months. Patient 13 received one prior line of therapy and achieved CR with uMRD after one cycle. He was successfully bridged to HSCT and remains in remission, with a follow-up duration of 26.74 months. Patients 7 and 11 had five and three prior lines of therapy, respectively, with NR, and died from the disease.

All five patients with a diagnosis of B-cell ALL were heavily pretreated with an average of 5.6 lines of prior therapy (median 6, range 3–8). Only patient 14 responded with CRi and uMRD after one cycle and was successfully bridged to HSCT after two cycles. She remains alive, with a follow-up of 13.4 months.

Genomic data, including cytogenetics and molecular diagnostics data, were available for all butone1 patient. As shown in Table 2, the patients displayed a very heterogeneous genomic landscape. Genomic alterations found in two or more patients, were analyzed, and are described here. Seven patients, all with a diagnosis of T-cell ALL/LBL, had NOTCH1 mutations. Of these, 71% (*n* = 5) achieved CR. Three patients harbored mutations in ASXL2, two of these patients had a diagnosis of B-cell ALL and had NR, and one patient had T-cell LBL and achieved CR. Two patients had a mutation in BCORL1, one with B-cell ALL who had NR, and one with T-cell LBL who achieved CR. Two patients harbored mutations in KDM6A, both with a diagnosis of T-cell ALL but only one patient had CR. Two patients with a mutation in CREBBP had NR, one with a diagnosis of B-cell ALL, and one with T-cell ALL. Two patients with B-cell ALL had a PAX5 mutation, one patient achieved CR, and one patient had NR. There was no clear correlation between any of the genomic alterations and the response to venetoclax.

## 4. Discussion

This single-institution retrospective review of pediatric/AYA patients treated with venetoclax for ALL/LBL displays increased evidence that venetoclax is safe in pediatric/ AYA patients. This cohort experienced the expected myelosuppression previously reported, but no unexpected toxicity when used in combination regimens. In addition, it builds upon evidence that venetoclax combination therapy is effective for both newly diagnosed and R/R pediatric T-cell ALL as well as T-cell LBL.

Preclinically, it has been shown that BCL-2 is more highly expressed in early thymic precursors and decreases with T-cell maturation/differentiation, making it reasonable that precursor T-cell ALL/LBL could be highly targeted by venetoclax [24]. Venetoclax has already been described as effective in adults with RR ALL, especially T-cell ALL, with particular attention paid to ETP ALL [26,31,32]. More recently, it was shown to be beneficial and safe in the pediatric population for myelodysplastic syndrome and AML, and there are emerging but sparse data on its use in lymphoid malignancies in pediatrics [27,33,34,35,36]. Pullarkat et al., in a study that combined venetoclax, navitoclax, and low-dose chemotherapy for RR lymphoblastic leukemia/lymphoma, demonstrated favorable results in 12 pediatric patients with ALL, with a 75% response rate [27]. Thus, what remains under-reported in pediatrics is the response to venetoclax used as upfront therapy for T-cell ALL/LBL and the response of R/R T-cell LBL to venetoclax.

Here, we report the largest population of pediatric/AYA patients and the largest reported pediatric population of T-cell LBL patients treated with venetoclax combination therapy. In our pediatric and young AYA cohort, we report on six patients with T-cell LBL and show that five of the six patients achieved CR after a median of one cycle. Three of the patients who achieved CR remain in remission, with a median follow-up time of 8.4 months. It is notable that of the three patients who remain in remission, two received venetoclax as upfront therapy, and this may have contributed to their favorable response. Results were similar for patients with a diagnosis of T-cell ALL. Patients with R/R T-cell LBL/ALL had median OS and PFS of 6 and 4.8 months, respectively. This is comparable to a previous report in the adult population that reported OS and PFS of 7.7 and 4 months, respectively [26], though it is important to note that the study in the adult population was primarily on T-cell ALL patients, with only one patient with T-cell LBL; therefore, is not directly comparable. CR2, which has historically been difficult to achieve in the R/R T-cell ALL/LBL population, was achieved by 75% (*n* = 6) of patients with R/R T-cell disease here, in comparison to 68% of patients with AALL07P1 [37]. Only one patient with B-cell ALL was able to achieve CR in our study, compared to 77% of patients with T-cell ALL/LBL who achieved CR/CRi.

Toxicity data in this pediatric and AYA population reiterated that myelosuppression, particularly grade 4 thrombocytopenia, was the primary side effect seen. It is important to note that, although the concurrent combination therapy did differ between patients, the majority received a traditional cytotoxic chemotherapy backbone, supporting the feasibility of venetoclax with conventional cytotoxic therapy backbone in the pediatric population. The common adverse event of myelosuppression was managed in all patients with either a decrease of venetoclax dosing to 100–200 mg/day or a shortened interval of 3–5 days in each cycle. Patients who received >7 days of consecutive therapy trended toward more significant thrombocytopenia with secondary delays in treatment.

This study should be viewed considering several limitations. Though this is the largest cohort of pediatric/AYA patients to received venetoclax for ALL/LBL, the sample size is still relatively small, with a median follow-up time of only 12.11 month. When patients are fractioned into cohorts of T-cell ALL, T-cell LBL, and B-cell ALL, these cohorts become smaller, with increased difficultly to draw generalizable conclusions about the therapeutic response.

Despite the limitations of a retrospective view, relatively small population size, and concurrent therapy differences, the sparsity of data currently in the literature on venetoclax use in pediatric ALL/LBL and the very poor outcomes for patients with relapsed T-cell ALL/LBL, make this review particularly important. This case series demonstrates that venetoclax should be considered as salvage chemotherapy in pediatric patients with RR lymphoblastic leukemia and lymphoma and should be investigated as upfront therapy for patients with T-cell lymphoblastic leukemias and lymphomas, malignancies that lack effective salvage therapies.

## 5. Conclusions

Given its clear activity, especially in the often difficult-to-treat RR T-cell ALL/LBL population, venetoclax should be strongly considered as an addition to frontline therapy for future pediatric studies. The combinations with nelarabine in upfront treatment in NCT00501826 and with navitoclax for RR disease are encouraging as potential combination therapy options. Overall, venetoclax appears to be safe and well tolerated in pediatric patients. Patients should be monitored closely for prolonged myelosuppression and febrile neutropenia. Further studies are needed to establish optimal dose, length of therapy, proper combination and to assess its long-term safety.

## Figures and Tables

**Table 1:** Baseline patient demographics and number of prior treatments.

Baseline Characteristics	Patients: *n* (%)
Age in years, median (range)	20 (6–21)
Sex	
Female	5 (28)
Male	13 (72)
Race	
Asian	5 (28)
Black	1 (5)
Hispanic	5 (28)
White	7 (39)
Diagnosis	
B-cell ALL	5 (28)
T-cell ALL	7 (39)
T-cell LBL	6 (33)
Prior Regimens	
0	4 (22)
1	6 (33)
>3	8 (44)
Previous Transplantation	
Yes	5 (28)
No	13 (72)

Baseline patients’ characteristics and number of regimens prior to venetoclax treatment and indication of whether the patients had a history of a prior hematopoietic stem cell transplant. Abbreviations: ALL—Acute lymphoblastic leukemia, LBL—Lymphoblastic lymphoma.

**Table 2:** Patient disease characteristics, concurrent therapy, dosing schedule for venetoclax, number of cycles given, response and toxicity.

Patient Number	Diagnosis	Age/Sex	Cytogenetics	NGS and PCR Mutation Findings	Number of Prior Therapies	Prior Therapy	Concurrent Chemotherapy	Dosing Schedule	Cycles	Response	Toxicity
1	T-cell LBL	12/M	Negative	Negative	1	AALL0434	HyperCVAD, Decitabine	100 mg/day (concurrent Posaconazole)	2	NR	Thrombocytopenia
2	T-cell LBL	20/M	Unknown	Unknown	4	AALL1231; NECTAR protocol; Clofarabine; Cytarabine/Mitoxantrone	Decitabine	400 mg/day	1	CR	Febrile neutropenia, thrombocytopenia, coagulopathy
3	T-cell LBL	20/M	2 extra copies of PDGFRB	Negative	1	HyperCVAD	HyperCVAD, nelarabine	400 mg/day	7	CR	Sepsis, pancreatitis
4	T-cell LBL	20/F	Negative	STAT5A, SH2B3, ASXL2, RUNX1, PHF6	0	None	HyperCVAD, nelarabine, pegASP	400 mg/day	4	CR	Thrombocytopenia, neutropenia
5	T-cell LBL	21/M	Negative	Negative	0	None	HyperCVAD, nelarabine	400 mg/day	3	CR	Thrombocytopenia
6	T-cell LBL	21/F	Negative	NOTCH1, TP53, BCORL1	1	HyperCVAD	Fludarabine, cytarabine, idarubicin, pegASP	400 mg/day	2	CR	Febrile neutropenia, sepsis, myelosuppression
7	T-cell ALL	21/M	TP53 deletion	NOTCH1, KDM6A, CREBBP	5	HyperCVAD; Nelarabine; MOAD; HSCT; MOAD	Nelarabine, etoposide, cyclophosphamide, decitabine	400 mg/day	1	NR	Pneumonia, sepsis, thrombocytopenia, hyperbilirubinemia
8	T-cell ALL	19/M	Negative	NOTCH1, IL7F	0	None	HyperCVAD	400 mg/day	13	CR	Thrombocytopenia, sepsis, hyperbilirubinemia
9	T-cell ALL	17/M	iAMP21, trisomy 8	NOTCH1, STAT5B	1	AALL0434	HyperCVAD	200 mg/day (concurrent voriconazole)	1	CRi	Febrile neutropenia, thrombocytopenia
10	ETP T-cell ALL	19/M	Negative	TCRG rearrangement	1	AALL0434; cyclophosphamide, cytarabine	Fludarabine, cytarabine, gemtuzumab, methotrexate	100 mg/day (concurrent voriconazole)	1.5	CR	Thrombocytopenia, neutropenia
11	T-cell ALL	18/M	t(4;6)	IL7R, NOTCH1, SF3A1, TCRB/G rearrangement	3	AALL0434; CALBG; HyperCVAD	HyperCVAD	400 mg/day	1	NR	Thrombocytopenia, neutropenia
12	T-cell ALL	21/M	Negative	NOTCH1, KDM6A	0	None	HyperCVAD, nelarabine, pegASP	400 mg/day	7	CR	Febrile neutropenia, thrombocytopenia
13	T-cell ALL	22/M	Negative	SUZ12, NOTCH1, FBXW7, KRAS, WT1, Deletion CDKN2A and P16	1	HyperCVAD	Nelarabine, pegASP, Gemtuzumab	100 mg/day	2	CR	none
14	B-cell ALL	21/F	One copy loss of FGFR1, one copy gain of CRLF2	PAX5, STAG2	5	ALL1131; HyperCVAD, rituximab, inotuzumab, blinatumomab; HSCT; blinatumomab, MTX, AraC; Kymriah	CVD	400 mg/day	2	CRi	Febrile neutropenia, thrombocytopenia
15	B-cell ALL	18/M	ETV6/RUNX1 rearrangement	Negative	6	ALLR3; HSCT; HyperCVAD; Decitabine; Sleeping Beauty CAR-T; Kymriah	CVD	100 mg/day (concurrent voriconazole)	1	NR	Thrombocytopenia, neutropenia
16	B-cell ALL	11/M	ETV6/RUNX1 fusion	ASXL2, ETV6, TP53	6	AALL0932; KITE; Kymriah; CD19/CD22 CAR; HSCT transplant; AALL0434	CVD	340 mg/day (360 mg/m^2^)	1	NR	Myelosuppression, hyperbilirubinemia
17	B-cell ALL	20/F	One copy loss of ABL1, ABL2, PDGFR, CRLF2; one copy gain of JAK2, ETV6, RUNX1, amplification of KMT2A	NF1, TP53, WT1	3	HyperCVAD, inotuzumab; fludarabine, cytarabine; CD22 CAR	HyperCVAD, rituximab	100 mg (concurrent voriconazole)	1	NR	Thrombocytopenia
18	B-cell ALL	6/F	Negative	ASXL2, BCORL1, CREBBP, DNMT3A, NF1, PAX5, FLT3, SF1, KMT2A	8	ALLR3 + Bortezomib; Kymriah; Blinatumomab; AALL1621; AALL1131; Nivolumab; vincristine/daunorubicin/MTX	CVD	70 mg/day (concurrent voriconazole)	1	NR	Thrombocytopenia, sepsis, hyperbilirubinemia

Patients’ baseline disease characteristics including age and sex of the patients at the time of venetoclax treatment, type of leukemia or lymphoma, cytogenetic anomalies, next-generation sequencing and polymerase chain reaction mutations, number and type of therapy regimens prior to venetoclax, concurrent therapy given and dosage of venetoclax, number of cycles of therapy given, response (complete, complete with incomplete blood count recovery, partial, or no response), and toxicity attributed to venetoclax. Abbreviations: NGS—next-generation sequencing. PCR—polymerase chain reaction. HyperCVAD—hyper-fractionated cyclophosphamide, vincristine, dexamethasone, doxorubicin, methotrexate, cytarabine. CVD—cyclophosphamide, vincristine, dexamethasone. MOAD—methotrexate, L-asparaginase, dexamethasone. HSCT—hematopoietic stem cell transplant. KITE—Yescarta CAR-T. NIH—National Institutes of Health. NECTAR Protocol—Nelarabine, etoposide, cyclophosphamide. CALBG—Cyclophosphamide, daunorubicin, vincristine, prednisone, asparaginase. ALLR3—Vincristine, Mitoxantrone/Idarubicin, dexamethasone, vincristine, PegASP—pegylated Asparaginase, cotrimoxazole. NR—no response. CR—complete remission. CRi—complete remission without blood count recovery. Safety profiles and toxicities.

**Table 3:** Adverse events attributable to venetoclax per CTCAE v5.0.

Adverse Event	≥Grade 3 *N* (%)	Grade 3	Grade 4
Thrombocytopenia	16 (89)	0	16
Neutropenia	10 (53)	0	9
Elevated bilirubin	4 (22)	4	0
Sepsis	5 (28)	4	1
Febrile neutropenia	5 (28)	2	3
Elevated AST/ALT	1 (5)	1	0
Pneumonia	1 (5)	0	1
Coagulopathy	1 (5)	0	1
Mucosal infection	1 (5)	1	0

Number of adverse events, grade 3 or 4, in patients undergoing venetoclax therapy. Abbreviations: AST/ALT—aspartate aminotransaminase/alanine aminotransferase.

**Table 4:** Summary of patient response to venetoclax combination therapy.

Response	CR/CRi # (%)	NR
Overall	11 (61)	7 (39)
Overall R/R disease	7 (50)	7 (50)
Overall T-cell disease (ALL/LBL)	10 (77)	3 (23)
R/R T-cell ALL/LBL	6 (75%)	2 (25)
Overall T-cell LBL	5 (83)	1 (17)
R/R T-cell LBL	3 (75)	1 (25)
Upfront T-cell LBL	2 (100)	0
Overall T-cell ALL	5 (71)	2 (29)
R/R T-cell ALL	3 (75)	1 (25)
Upfront T-cell ALL	2 (100)	0
Overall B-cell ALL	1 (20)	4 (80)
R/R B-cell ALL	1 (20)	4 (80)
Upfront B-cell ALL	-	-

Patients’ response rate to venetoclax combination therapy in relation to disease type and relapsed/refractory disease and upfront combination therapy.

## Data Availability

Available upon request from corresponding author.
